# Supplementary material for: Learning to detect AI texts and learning the limits
Source: PLoS One. 2025 Oct 15;20(10):e0333007. doi: 10.1371/journal.pone.0333007 (PMC12527182; doi:10.1371/journal.pone.0333007)
Supplement: S2 File — All data and scripts used in the study, some additional descriptive statistics, scalable formate figures. (ZIP) [file pone.0333007.s002.tgz › data and scripts/OSF/scripts/analysis/Dependencies/GLS1_distance_vs_Reaction_time_log.pdf]

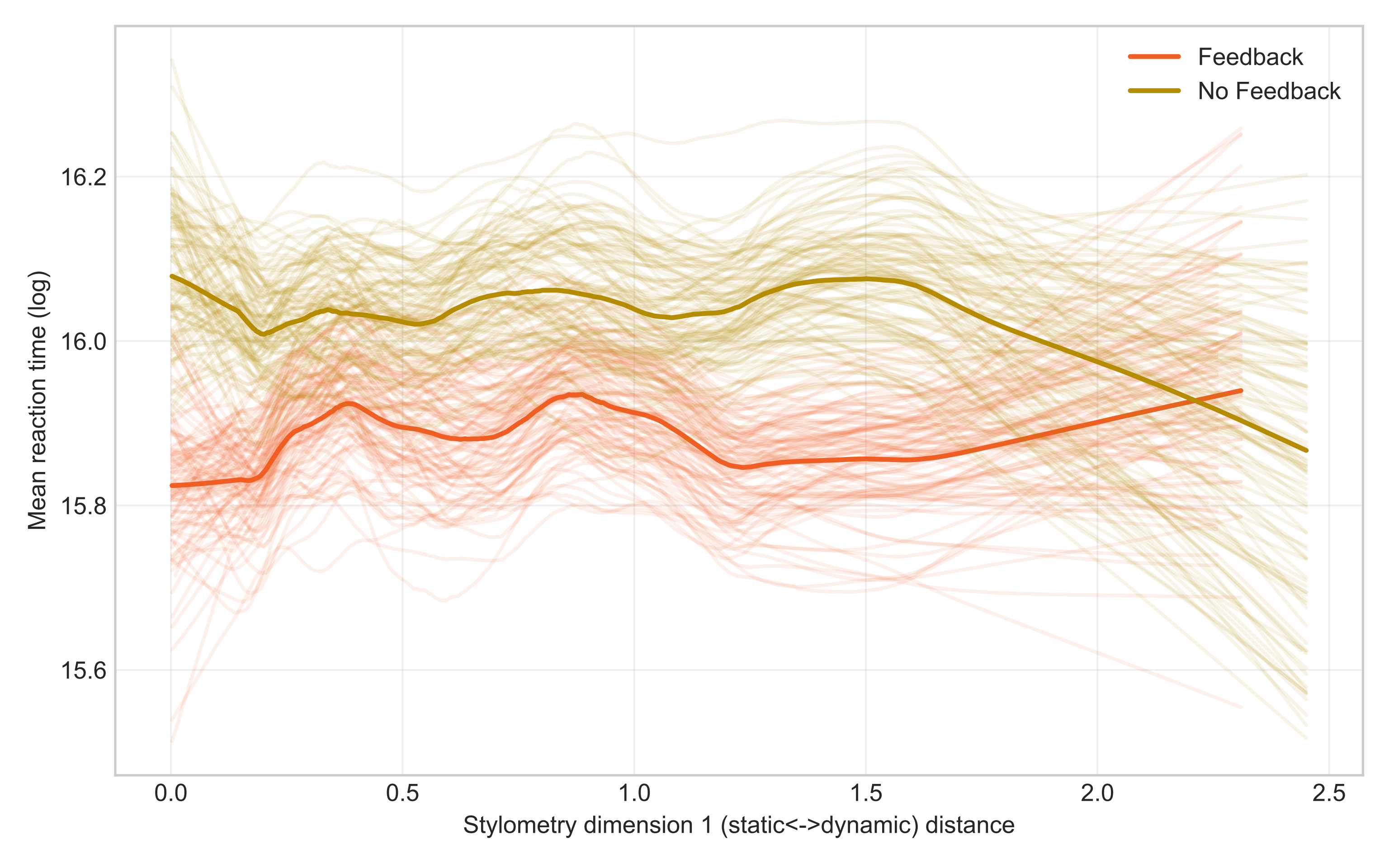
Feedback
No Feedback
Mean reaction time (log)
16.2
16.0
15.8
15.6
0.0
0.5
1.0
1.5
2.0
2.5
Stylometry dimension 1 (static<->dynamic) distance
